# Peritoneal Dialysate Glucose Load and Systemic Glucose Metabolism in Non-Diabetics: Results from the GLOBAL Fluid Cohort Study

**DOI:** 10.1371/journal.pone.0155564

**Published:** 2016-06-01

**Authors:** Mark Lambie, James Chess, Jun-Young Do, Hyunjin Noh, Hi-Bahl Lee, Yong-Lim Kim, Angela Summers, Paul Ford Williams, Sara Davison, Marc Dorval, Nick Topley, Simon John Davies, on behalf of Global Fluid Study Investigators

**Affiliations:** 1 Institute of Applied Clinical Research, Keele University, Stoke on Trent, United Kingdom; 2 Institute of Nephrology, Cardiff University School of Medicine, Heath Park, Cardiff, United Kingdom; 3 Renal Unit, Morriston Hospital, Swansea, United Kingdom; 4 Division of Nephrology, Yeungnam University Hospital, Daegu, South Korea; 5 Hyonam Kidney Laboratory, Soon Chun Hyang University, Seoul, South Korea; 6 Division of Nephrology and Clinical Research Center for ESRD in Korea, Kyungpook National University Hospital, Daegu, South Korea; 7 Manchester Institute of Nephrology and Transplantation, Manchester Royal Infirmary, Manchester, United Kingdom; 8 Ipswich Hospital NHS Trust Ipswich Hospital, Heath Road, Ipswich, United Kingdom; 9 Division of Nephrology and Immunology, University of Alberta, Edmonton, Canada; 10 Division of Nephrology, Dr. Georges-L.-Dumont University Hospital Centre, Moncton, New Brunswick, Canada; Mexican Social Security Institute, MEXICO

## Abstract

**Background and Objectives:**

Glucose control is a significant predictor of mortality in diabetic peritoneal dialysis (PD) patients. During PD, the local toxic effects of intra-peritoneal glucose are well recognized, but despite large amounts of glucose being absorbed, the systemic effects of this in non-diabetic patients are not clear. We sought to clarify whether dialysate glucose has an effect upon systemic glucose metabolism.

**Methods and Materials:**

We analysed the Global Fluid Study cohort, a prospective, observational cohort study initiated in 2002. A subset of 10 centres from 3 countries with high data quality were selected (368 incident and 272 prevalent non-diabetic patients), with multilevel, multivariable analysis of the reciprocal of random glucose levels, and a stratified-by-centre Cox survival analysis.

**Results:**

The median follow up was 5.6 and 6.4 years respectively in incident and prevalent patients. On multivariate analysis, serum glucose increased with age (β = -0.007, 95%CI -0.010, -0.004) and decreased with higher serum sodium (β = 0.002, 95%CI 0.0005, 0.003) in incident patients and increased with dialysate glucose (β = -0.0002, 95%CI -0.0004, -0.00006) in prevalent patients. Levels suggested undiagnosed diabetes in 5.4% of prevalent patients. Glucose levels predicted death in unadjusted analyses of both incident and prevalent groups but in an adjusted survival analysis they did not (for random glucose 6–10 compared with <6, Incident group HR 0.92, 95%CI 0.58, 1.46, Prevalent group HR 1.42, 95%CI 0.86, 2.34).

**Conclusions:**

In prevalent non-diabetic patients, random glucose levels at a diabetic level are under-recognised and increase with dialysate glucose load. Random glucose levels predict mortality in unadjusted analyses, but this association has not been proven in adjusted analyses.

## Introduction

There is a large amount of laboratory and clinical evidence of glucose-based peritoneal dialysate causing significant damage to the peritoneal membrane [1,2] but there have been far fewer studies documenting the systemic consequences of glucose-based dialysate. Significant glucose absorption from the peritoneum does occur during peritoneal dialysis (PD), such that glucose induced hyperosmolarity precludes the use of dialysis solutions with very high glucose concentrations. [3]

Insulin resistance, along with hypertriglyceridaemia, low HDL-cholesterol, hypertension and abdominal obesity, are defined as metabolic syndrome (MetS), [4,5] a condition thought to be related to sustained high sugar intake in the general population [6] and which predicts cardiovascular mortality. [7] Impaired fasting glucose increases during PD by up to 49.8%, along with other features of MetS. [8**]** All the features of MetS have been associated with dialysate glucose exposure except for impaired fasting glucose, but this was related to prior dialysate glucose exposure rather than a contemporaneous measure. Impaired fasting glucose predicts mortality in the general population,[7] and high glucose levels in PD patients are associated with mortality on univariable analysis [9] so whether a reduction in dialysate glucose exposure can mitigate the increase in hyperglycaemia is an important clinical question.

We hypothesised that a contemporaneous measure of dialysate glucose loading would be associated with systemic glucose levels, and that impaired glucose homeostasis would predict mortality in a fully adjusted analysis of non-diabetic patients. We used the GLOBAL Fluid Study cohort to address these questions.

## Methods and Materials

### Study design

The study has been described in detail elsewhere [10] but in brief, the Global Fluid Study is an international, multicentre, prospective cohort study of incident and prevalent patients commenced in 2002. Eligible patients were any PD patients over the age of 18 providing informed consent. Incident patients were defined as first data collection time point within the first 90 days of PD. Follow up was censored in December 2011. Ten centres were selected based on the highest quality existing data then iteratively checked to optimise final data completeness, and a cross-section of all non-diabetic patients from these units was used for this analysis at the point of study entry. Despite this process, one centre had significantly worse data quality in the final analysis, so sensitivity analyses excluding this centre were pre-specified.

Ethical approval was obtained from the Multi-Centre Research Ethics Committee for Wales covering the United Kingdom, from Kyungpook National University Hospital Ethics Committee covering Korea and from University of Alberta Ethics Committee covering Canada. Written informed consent was obtained from all patients.

### Data collection

All clinical data were recorded on a custom built database (PDDB). Demography was recorded and comorbidity was assessed with the validated Stoke comorbidity index. This included the diagnosis of diabetes which was recorded from routine clinical data at the centre. Routine blood tests, including albumin and random glucose, were performed locally and, if necessary, converted into the same units. Data was not available on the exact timing of the sample. The samples of dialysate and serum taken at the first assessment within the study were assayed for IL-6 by electrochemiluminescence.

PD related measurements included residual renal function, dialysis regime and dose, and peritoneal membrane function using the peritoneal equilibration test (solute transport rate: dialysate to serum creatinine ratio (PSTR) and net UF capacity at 4 hours with 2.27% or 3.86% glucose). The Daily Dialysate Glucose (DDG) exposure was calculated as total grammes of unhydrated glucose within the 24 hour dialysate regime as recorded on the day of assessment (e.g. 2 litres of 1.36% glucose based dialysate = 2 x 13.6 grammes = 27.2 grammes).

### Statistical analysis

Comparisons between glucose categories were made with one-way ANOVA, Kruskal-Wallis or chi-squared tests depending on the variable.

Missing data were considered missing at random and complete case analysis was used. Pre-specified sensitivity analyses excluding one centre with the highest level of missing data were performed.

Random glucose levels were expected to deviate significantly from a normal distribution therefore transformations on the ladder of powers were tested, with the reciprocal transformation demonstrating a normal distribution. Multivariable multilevel, random intercept models, which account for centre effects, were used to explore determinants of random glucose. Significance testing was by the Wald test. The Iterative Generalised Least Squares method was used for coefficient estimation.

Log-rank tests were used for univariable, and Cox models stratified by centre with robust standard errors were used for multivariable, survival analysis. To allow for non-linearity, glucose levels were categorised into <6 mmol/l, 6-10mmol/l and >10 mmol/l; the levels chosen to aid biological interpretability whilst maintaining group size. The >10mmol/l group was defined to include patients who were undiagnosed diabetics based on random glucose levels >11.1mmol/l or were close to this, leaving the intermediate 6-10mmol/l group more reliably composed of non-diabetics. A secondary analysis with glucose included as a continuous variable in the same adjusted Cox model was also performed.

MLWin 2.26 [11] was used via runmlwin for multilevel regression and StataIC 12 (StataCorp LP, College Station, TX) for the other calculations.

## Results

### Patient details

Demographic and clinical data are shown in Table 1. There were 576 incident patients, with glucose available in 548, of whom 327 were non-diabetic in whom there were 116 deaths during a median follow up of 5.93 years. There were 384 prevalent patients, with glucose available in 360, of whom 242 were non-diabetic with 96 deaths during a median follow up of 7.29 years. To aid comparisons with the differing definitions used in other studies we have also provided the frequency of glucose levels according to these different definitions. In incident patients, glucose levels were ≥6.2 mmol/l in 29.8%, 7.0–11.1mmol/l in 15.3% and >11.1mmol/l in 3.7%, and in prevalent patients they were ≥6.2 mmol/l in 32.6%, 7.0–11.1mmol/l in 14.0% and >11.1mmol/l in 5.4%. Outcome data were unavailable for 8 prevalent patients from one centre and 16 incident patients (15 from the same centre), but sensitivity analyses excluding this centre made no difference.

**Table 1:** Patient Demographics. p value represents differences between glucose categories, in bold for p<0.05.

	Incident					Prevalent				
	All	Glucose <6 mmol/l	Glucose 6–10 mmol/l	Glucose >10 mmol/l	p value	All	Glucose <6 mmol/l	Glucose 6–10 mmol/l	Glucose >10 mmol/l	p value
**Number**	327	219	95	13		242	151	74	17	
**Age (years)**	55.1 (17.0)	52.7 (17.4)	59.9 (15.6)	59.7 (12.2)	**0.002**	53.3 (15.8)	52.0 (16.2)	55.3 (15.5)	56.5 (12.8)	0.1
**Female Gender**	34.3%	36.2%	40.6%	46.2%	0.4	47.9%	49.7%	47.3%	35.9%	0.5
**Korean**	37.4%	33.0%	42.6%	76.9%	**0.002**	34.7%	29.1%	41.9%	52.9%	0.1
**Duration of PD (median, days)**	38	36 (22–52)	42 (29.5–51.5)	36 (28.5–51)	0.1	369 (177–819)	377 (193–818)	358 (162–716)	371 (167–605)	0.7
**BMI (kg/height**^**2**^**)**	24.8 (4.8)	24.6 (4.9)	25.3 (4.7)	23.9 (3.8)	0.4	24.6 (4.4)	24.4 (4.0)	25.3 (4.9)	22.9 (3.9)	0.1
**Blood pressure (mmHg)**	135/81 (20/12)	135/82 (20/12)	136/79 (22/13)	137/80 (24/14)	0.6/0.3	134/82 (21/13)	135/82 (22/14)	132/81 (17/10)	136/83 (23/13)	0.6/0.7
**4 hour PSTR (D/P Cr)**	0.69 (0.12)	0.69 (0.13)	0.69 (0.10)	0.71 (0.13)	0.9	0.70 (0.11)	0.71 (0.12)	0.70 (0.11)	0.73 (0.13)	0.5
**Albumin (g/l)**	36.1 (5.0)	36.5 (5.0)	35.5 (4.7)	34.2 (5.7)	0.1	36.1 (4.7)	36.7 (4.4)	35.1 (4.7)	33.9 (5.6)	**0.005**
**Urine volume (median, litres)**	0.9 (0.48–1.52)	0.99 (0.50–1.70)	0.85 (0.49–1.21)	0.6 (0.20–1.24)	0.2	0.52 (0.15–1.13)	0.57 (0.20–1.21)	0.55 (0.14–1.15)	0.3 (0.02–0.93)	0.3
**Comorbidity (Low/Intermediate/High)**	56.8, 31.0, 12.3%	57.3, 39.9, 2.8%	56.4, 41.5, 2.1%	53.8, 38.5, 7.7%	0.95	62.8, 34.3, 2.9%	64.9, 32.5, 2.6%	60.8, 35.1, 4.1%	52.9, 47.1, 0%	0.8
**Serum IL-6 (median, pg/ml)**	1.27 (0.55–2.75)	1.2 (0.50–2.55)	1.73 (0.66–3.13)	1.75 (0.74–2.74)	0.1	1.1 (0.58–2.00)	0.94 (0.54–1.62)	1.51 (0.82–2.71)	0.95 (0.71–1.51)	**0.004**
**Total Daily Dialysate Glucose (grammes/day)**	120.8 (36.8)	123.8 (41.2)	114.5 (25.9)	117.6 (20.4)	0.2	132.0 (45.8)	128.7 (38.9)	136.2 (50.0)	142.7 (74.4)	0.3
**Total dialysate volume (litres)**	7.98 (1.28)	7.97 (1.47)	8.02 (0.86)	7.93 (0.25)	0.8	8.35 (1.95)	8.37 (2.06)	8.32 (1.83)	8.29 (1.57)	0.98
**Biocompatible solution usage**	23.8%	25.3%	18.1%	38.5%	0.2	16.2%	16.7%	13.5%	23.5%	0.6
**Icodextrin solution usage**	14.8%	15.2%	16.0%	0%	0.3	23.8%	25.5%	23.0%	11.8%	0.5
**Use of APD**	6.5%	8.4%	3.2%	0%	0.09	15.8%	16.7%	15.1%	11.8%	0.9

### Factors affecting glucose

**Fig 1:**
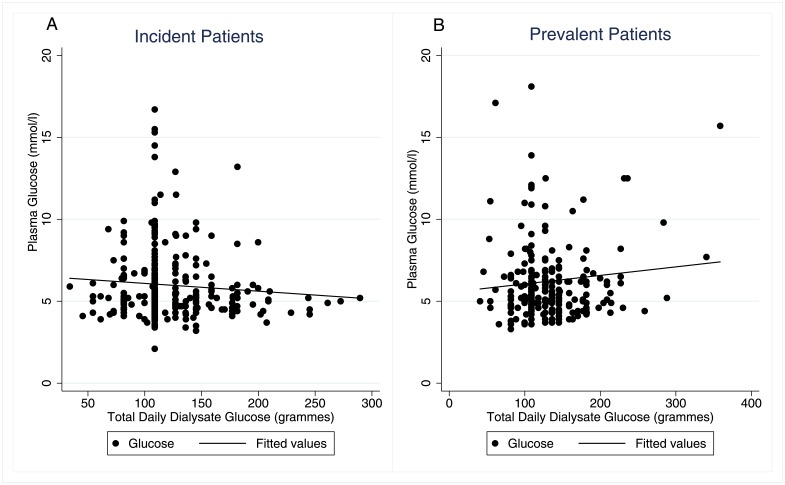
Scatterplot of Serum Glucose vs. Total Daily Dialysate Glucose in Non-Diabetic Patients. The line represents a univariable linear regression. A—Incident patients, B—Prevalent patients.

**Fig 2:**
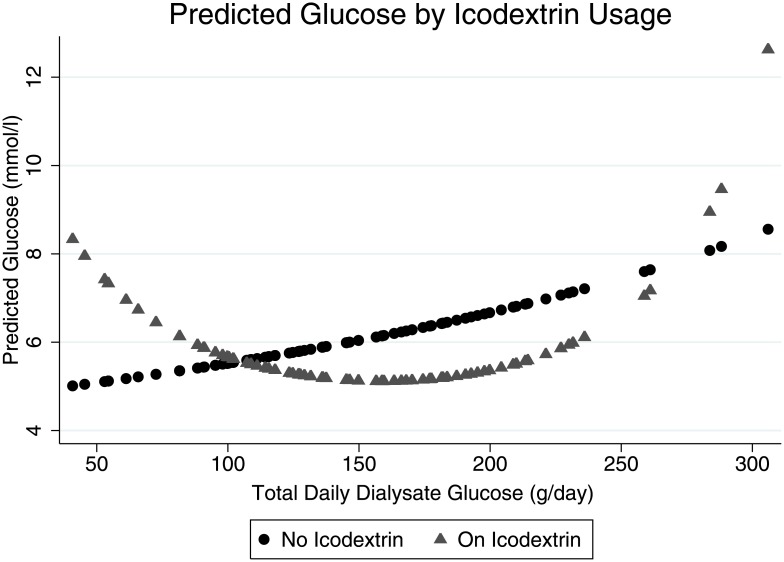
Predicted Glucose Levels by Icodextrin Usage.

For incident patients, DDG load did not predict glucose levels on unadjusted (β = -0.00002, 95% CI (-0.00013, 0.00018), p = 0.8) or adjusted analysis (Fig 1A and Table 2). On both unadjusted (β = -0.003, 95% CI (-0.005, -0.001), p = 0.001) and on adjusted analysis (Fig 1B and Table 2), the DDG load predicted random glucose levels in prevalent patients. Sensitivity analyses excluding one centre with higher levels of missing data affected the results in prevalent patients in Table 2, strengthening the association with DDG and weakening it with albumin. We also tested for 3 pre-specified interactions—between DDG and peritoneal solute transport rate, DDG and the type of PD (automated PD versus continuous ambulatory PD), and DDG and Icodextrin usage. The first 2 had no significant effect so were not included, but there was a significant interaction with Icodextrin usage in prevalent patients best modelled as a quadratic term (Table 2). Predicted values from the regression model on or off Icodextrin are shown in Fig 2, demonstrating that for patients on Icodextrin there was a U-shaped relationship between dialysate glucose and serum levels. As the association with serum sodium may have been an effect of glucose levels rather than vice versa, a sensitivity analysis excluding serum sodium was performed but made no substantive difference to the results (S1 Table).

**Table 2:** Predictors of the reciprocal of random serum glucose levels. Reciprocal of glucose (1/mmol/l) used as dependent variable in multilevel, multivariable model. Significant results (p<0.05) marked in bold.

	Incident		Prevalent	
	β Coefficient (95% CI)	p value	β Coefficient (95% CI)	p value
**Daily Dialysate Glucose**	0.00014 (-0.00001, 0.00029)	0.07	**-0.00029** (-0.00045, -0.00013)	**0.001**
**Korean**	**-0.027** (-0.053, -0.001)	**0.04**	-0.010 (-0.049, 0.028)	0.6
**BMI**	-0.00088 (-0.00199, 0.00023)	0.1	-0.00063 (-0.00200, 0.00074)	0.4
**Age (per year)**	**-0.00070** (-0.00102, -0.00038)	**<0.001**	**-0.00039** (-0.00077, -0.00001)	**0.04**
**Gender**	-0.0066 (-0.0174, 0.0041)	0.2	-0.0055 (-0.0167, 0.0057)	0.3
**Systolic BP (per 10)**	-0.0011 (-0.0035, 0.0014)	0.4	0.0022 (-0.0005, 0.0048)	0.1
**Peritoneal Solute Transport Rate**	0.038 (-0.012, 0.088)	0.1	-0.020 (-0.075, 0.035)	0.5
**Duration of PD**	-0.067 (-0.166, 0.031)	0.2	-0.00031 (-0.00360, 0.00298)	0.9
**Albumin**	0.00019 (-0.00103, 0.00142)	0.8	0.0010 (-0.0004, 0.0024)	0.1
**Serum Sodium**	**0.0020** (0.0006, 0.0034)	**0.004**	0.0016 (-0.0001, 0.0033)	0.06
**Serum IL-6**	-0.0014 (-0.0185, 0.0157)	0.9	-0.0099 (-0.0338, 0.0140)	0.4
**Urine Volume**	0.0030 (-0.0046, 0.0106)	0.4	-0.0013 (-0.0122, 0.0096)	0.8
**Comorbidity**	-0.00033 (-0.00654, 0.00720)	0.9	0.0041 (-0.0027, 0.0109)	0.2
**Icodextrin**	0.0079 (-0.0072, 0.0231)	0.3	-0.15 (-0.24, -0.07)	**0.001**
Icodextrin*Dialysate Glucose Icodextrin*Dialysate Glucose^2			0.0020 (0.0008, 0.0033) 0.0000053 (-0.0000095, -0.0000012)	**0.001 0.01**

### Effect of glucose on mortality

**Fig 3:**
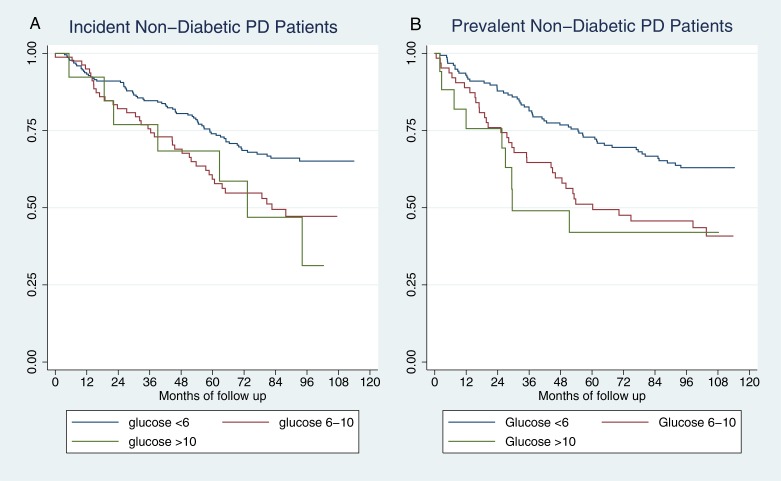
Kaplan-Meier plots of survival by random glucose in non-diabetic PD patients. A—Incident patients, B—Prevalent patients.

Kaplan-Meier plots of survival in non-diabetic patients by random glucose categories are shown in Fig 3, and they both demonstrated a significant difference on unadjusted analysis. Table 3 shows the results of adjusted Cox models for the same groups, whereby the effect of glucose on mortality is completely removed by adjustment in incident patients. In prevalent patients there is still a trend towards higher mortality but it is not statistically significant (HR’s for glucose 6-10mmol/l 1.45, 95% CI 0.90–2.36, for >10mmol/l 1.32, 95% CI 0.54–3.27, both compared with <6mmol/l).

**Table 3:** Predictors of mortality.

	Incident Patients		Prevalent Patients	
	Hazard Ratio (95% CI)	p value	Hazard Ratio (95% CI)	p value
**Age (per decade)**	1.92 (1.51–2.41) **	<0.001	1.78 (1.48–2.14)**	<0.001
**Serum IL-6 (per log order)**	3.07 (1.70–5.54)**	<0.001	3.08 (1.22–7.79)*	0.02
**Albumin**	0.96 (0.92–0.999)*	0.049	0.97 (0.93–1.02)	0.3
**PSTR (per 0.1 increased D/P Cr)**	1.05 (0.91–1.21)	0.5	1.24 (1.02–1.50)*	0.03
**Duration of PD (per month)**	1.14 (0.70–1.85)	0.6	1.013 (1.003–1.023)*	0.01
**RRF (per litre of urine volume)**	0.80 (0.60–1.08)	0.1	0.65 (0.41–1.03)	0.06
**Comorbidity**	1.48 (1.18–1.86)**	0.001	1.39 (1.15–1.69)**	0.001
**Glucose (ref ≤6) Glucose >6, <10 Glucose ≥10**	0.95 (0.59–1.51), 1.15 (0.58–2.29)	0.8, 0.7	1.45 (0.90–2.36), 1.32 (0.54–3.27)	0.13, 0.5

*p<0.05,

**p<0.01

In a sensitivity analysis, glucose was included as a continuous variable in the same adjusted model and it was not statistically significant for incident patients (HR 1.0002 for 1mmol/l increase, 95% CI (0.97, 1.03), p = 0.993) or for prevalent patients (HR = 1.035 for 1mmol/l increase, 95% CI (0.96, 1.11), p = 0.4).

### Missing Data

Missing data ranged from 0 to 4.8% for different variables. Loss to follow-up was trivial, with 7 and 5 cases in the incident and prevalent groups respectively.

## Discussion

This is the first study to demonstrate an effect of dialysate prescription on systemic glucose metabolism in non-diabetic patients, with higher random glucose levels correlating with increasing dialysate glucose exposure. We have also replicated the finding of unadjusted higher mortality rates with higher serum glucose levels in a more widely generalisable population than shown previously, [9] although this association was not statistically significant in a more fully adjusted analysis than has been possible previously.

The American Diabetic Association (ADA) defines impaired glucose metabolism as either impaired fasting glucose or impaired glucose tolerance.[12] This makes the interpretation of all studies on this subject in PD patients limited by the glucose absorption that occurs during PD because, as the level of absorption is not usually known at the point of sample acquisition, it cannot be truly considered fasting unless PD is withheld, and a glucose tolerance test requires a defined enteral dose. However, large studies necessary to detect subtle effects require straightforward tests. Our study used a pragmatic solution of completely random glucose levels whilst other studies opted for orally but not peritoneally fasted glucose levels. [8,9] The diagnostic criteria for impaired fasting glucose or impaired glucose tolerance in the general population cannot be assumed to hold for either of these approaches in PD patients.

In one study of non-diabetic Chinese patients 49.8% developed impaired fasting glucose (>6.2mmol/l) during PD [8] whereas in another 19.0% developed impaired fasting glucose (7.0–11.1mmol/l) after 1 month of PD. [9**]** Our study found a much lower rate of glucose levels >6.2mmol/l compared to the former study, and results similar to, but lower than, those from the latter study. This was despite our patients not being orally fasted whilst the other studies with higher rates were orally fasted. Taken together these rates imply that oral fasting does not have a large impact on the glucose levels in PD patients if they are not peritoneally fasted as well.

The very high concentrations of dialysate glucose used in the 1960’s caused high serum glucose levels, [3] but whether the concentrations currently used in dialysate have such an effect is not known. One study found PD patients had a similar but lower incidence of post-transplant diabetes than haemodialysis patients, [13] whilst another more recent study found that PD patients develop less de novo diabetes than HD patients. [14] These studies both suggest that dialysate glucose exposure does not greatly affect glucose metabolism, although it is associated with other aspects of MetS. [8] In this study by Jiang et al, systemic glucose levels were not associated with dialysate glucose, possibly as they correlated cumulative dialysate glucose exposure with subsequent glucose levels rather than the prescription at the time of glucose measurement. Our study used simultaneous measurements of dialysate glucose exposure and systemic levels to demonstrate this association in a mix of British, Canadian and Korean (as opposed to the previous studies of purely Chinese) prevalent patients. The difference in this association between incident and prevalent patients is likely to be due to the sustained glucose loading prevalent patients have undergone although theoretically it could be due to informative censoring.

Both our study and that of Szeto et al [9] suggest that undiagnosed diabetes is an issue in PD patients. The ADA diagnostic criteria [12] use casual glucose levels >11.1mmol/l with suggestive symptoms to diagnose diabetes and neither of these 2 studies had details of symptoms but Szeto found 8.3% in an incident group [9] and we found 3.7% and 5.4% in incident and prevalent groups respectively to have levels compatible with undiagnosed diabetes.

In line with other results,[9] age was predictive of glucose levels in patients after 1 month of PD but serum sodium also had a strong negative association with glucose levels. An association between hyponatraemia and hyperglycaemia is well recognised in other patient groups, and is thought to be due primarily to glucose-associated osmotic pressure causing a dilutional effect.[15] This might explain some of the association with comorbidity, inflammation and albumin found by Szeto.[9]

Ethnicity is a strong risk factor for the development of type II diabetes, with white Caucasians having a lower risk than Asians, Hispanics and Afro-Caribbeans.[16**]** This is commensurate with our observation in the incident group of Koreans having higher glucose levels than the predominantly white Caucasian reference population and may partly explain the higher glucose levels in the study by Szeto et al.[9]

The peritoneal solute transport rate is one of the potential determinants of the systemic effects of dialysate glucose, through modification of glucose absorption. We found no effect, possibly because the difference in absorption was relatively minor in comparison to the total amount absorbed; However, this study used a dialysate/serum creatinine ratio as the measure of peritoneal solute transport rate. The D/D0 glucose may be a better measure for this analysis but was not available.

Another important clinical question is whether it matters how the dialysate is prescribed. We tested for this by including the type of PD (APD vs CAPD) both as a main effect and an interaction and found no effect. This suggests that how the dialysate glucose is prescribed matters less than the amount that is prescribed.

Icodextrin absorption leads to increased plasma maltose levels [17] but does not cause glucose absorption in PD patients,[18] and this has been shown to improve glycemic control in diabetic PD patients. [19,20] It is therefore very unlikely to increase glucose levels in non-diabetic patients. There was, however, an interaction with DDG in prevalent patients which was highly significant. For patients on Icodextrin the predicted glucose initially declined with increased DDG but subsequently rose. Indication bias could explain this, if an abnormality of glucose metabolism not formally diagnosed as diabetes was recognised and treated with Icodextrin usage and glucose minimisation. The same relationship between increased DDG and glucose levels found in patients not on Icodextrin could explain the subsequent rise in glucose levels with increased DDG for patients on Icodextrin.

The survival analysis has confirmed the results from Szeto et al, with a significant unadjusted effect on mortality of serum glucose levels, but we extended this into an adjusted analysis. In incident patients there is no significant independent effect on mortality but it remains unclear whether there is an effect in prevalent patients, when dialysate glucose has its greatest effect, as the study was not specifically powered to pick up a hazard ratio as small as that estimated in our analysis. One study with 15 events did find a small effect of glucose levels on mortality on adjusted analysis. [21] A larger study using a more sensitive marker of glucose metabolism is required to investigate this further.

In the absence of larger studies using better markers of glucose metabolism, clear guidance on the safe dose of dialysate glucose is not possible. This study provides more evidence in support of minimising dialysate glucose exposure and suggests that, particularly if larger glucose doses are being used, systemic glucose metabolism should be monitored.

Limitations of this study include incomplete information on the presence of metabolic syndrome as the study was not primarily designed to investigate glucose metabolism. Glucose levels were measured locally although our use of a multilevel model adjusting for centre effects should account for problems arising from this. The study did not contain sufficiently reliable information on PD regimes to investigate the effect of different regimes rather than total dialysate glucose exposure. Whilst the study used 10 centres from 3 countries to ensure good generalisability, a degree of selection bias cannot be excluded as the selected centres had better data quality. As an observational study, causality cannot be proven although the association between dialysate glucose load and systemic levels fulfil most of the Bradford-Hill criteria for causality.[22] We have used a cross-sectional design but the difference between incident and prevalent groups would be better explored with longitudinal data. Using glucose levels >11.1mmol/l as the criteria, a small number of diabetics were included (although this in itself is informative), but as they were undiagnosed they were almost certainly not on treatment so the relationship between dialysate glucose and systemic effects should not be significantly affected.

Dialysate glucose load appears to have a significant effect on systemic glucose levels in non-diabetics and the effects are under-recognised. This should be factored in when prescribing peritoneal dialysate and increased awareness of the potential problems is necessary.

## Supporting Information

S1 Table Sensitivity Analysis for Determinants of Plasma Glucose Excluding Serum Sodium. (DOCX)
