# Supplementary material for: Peritoneal Dialysate Glucose Load and Systemic Glucose Metabolism in Non-Diabetics: Results from the GLOBAL Fluid Cohort Study
Source: PLoS One. 2016 Jun 1;11(6):e0155564. doi: 10.1371/journal.pone.0155564 (PMC4889040; doi:10.1371/journal.pone.0155564)
Supplement: S1 Table — (DOCX) [file pone.0155564.s001.docx]

## **Supplementary Material**

Table S1: Sensitivity Analysis for Determinants of Plasma Glucose Excluding Serum Sodium

|  | **Incident** | | **Prevalent** | |
| --- | --- | --- | --- | --- |
|  | **Coefficient (95% CI)** | **p value** | **Coefficient (95% CI)** | **p value** |
| **Daily Dialysate Glucose** | 0.00013 (-0.00003, 0.00028) | 0.1 | **-0.00027** (-0.00044, -0.00011) | **0.001** |
| **Korean** | **-0.031** (-0.060, -0.003) | **0.03** | **-**0.011 (-0.053, 0.031) | 0.6 |
| **BMI** | -0.00084 (-0.00195, 0.00026) | 0.1 | -0.00054 (-0.00190, 0.00082) | 0.4 |
| **Age (per year)** | **-0.00075** (-0.00107, -0.00043) | **<0.001** | -0.00031 (-0.00068, 0.00006) | 0.1 |
| **Male Gender** | -0.0051 (-0.0160, 0.0057) | 0.4 | -0.0030 (-0.0142, 0.0082) | 0.6 |
| **Systolic BP (per 10mmHg)** | -0.0011 (-0.0036, 0.0013) | 0.4 | 0.0011 (-0.0016, 0.0038) | 0.4 |
| **Peritoneal Solute Transport Rate** | 0.041 (-0.009, 0.090) | 0.1 | -0.027 (-0.082, 0.028) | 0.3 |
| **Duration of PD (per year)** | -0.072 (-0.172, 0.027) | 0.2 | -0.00028 (-0.00363, 0.00308) | 0.9 |
| **Albumin** | 0.0002 (-0.0010, 0.0014) | 0.7 | 0.0013 (-0.0001, 0.0027) | 0.1 |
| **Plasma IL-6** | -0.0036 (-0.0203, 0.0142) | 0.8 | -0.009 (-0.033, 0.015) | 0.5 |
| **Urine Volume** | 0.0036 (-0.0040, 0.0112) | 0.4 | 0.002 (-0.084, 0.013) | 0.7 |
| **Comorbidity** | -0.0016 (-0.0053, 0.0085) | 0.6 | 0.0040 (-0.0029, 0.0108) | 0.3 |
| **Icodextrin** | 0.0041 (-0.0110, 0.0192) | 0.3 | -0.105 (-0.187, -0.022) | **0.01** |
| **Icodextrin*Dialysate Glucose Icodextrin*Dialysate Glucose^2** |  |  | 0.0014 (0.0002, 0.0026) -0.0000039 (-0.0000079, -0.0000001) | **0.02** 0.06 |
